# Effect of 48 h Fasting on Autonomic Function, Brain Activity, Cognition, and Mood in Amateur Weight Lifters

**DOI:** 10.1155/2016/1503956

**Published:** 2016-11-29

**Authors:** Rima Solianik, Artūras Sujeta, Asta Terentjevienė, Albertas Skurvydas

**Affiliations:** Institute of Sports Science and Innovations, Lithuanian Sports University, Sporto Str. 6, Kaunas, Lithuania

## Abstract

*Objectives.* The acute fasting-induced cardiovascular autonomic response and its effect on cognition and mood remain debatable. Thus, the main purpose of this study was to estimate the effect of a 48 h, zero-calorie diet on autonomic function, brain activity, cognition, and mood in amateur weight lifters.* Methods.* Nine participants completed a 48 h, zero-calorie diet program. Cardiovascular autonomic function, resting frontal brain activity, cognitive performance, and mood were evaluated before and after fasting.* Results.* Fasting decreased (*p* < 0.05) weight, heart rate, and systolic blood pressure, whereas no changes were evident regarding any of the measured heart rate variability indices. Fasting decreased (*p* < 0.05) the concentration of oxygenated hemoglobin and improved (*p* < 0.05) mental flexibility and shifting set, whereas no changes were observed in working memory, visuospatial discrimination, and spatial orientation ability. Fasting also increased (*p* < 0.05) anger, whereas other mood states were not affected by it.* Conclusions.* 48 h fasting resulted in higher parasympathetic activity and decreased resting frontal brain activity, increased anger, and improved prefrontal-cortex-related cognitive functions, such as mental flexibility and set shifting, in amateur weight lifters. In contrast, hippocampus-related cognitive functions were not affected by it.

## 1. Introduction

Many religions and cultures incorporate fasting into their rituals. Fasting is also widely used and recommended for rapid weight loss [1–3] and for disease prevention and treatment [2]. It is well known that long-term intermittent and periodic fasting promotes health and reduces the risk of many chronic diseases, such as cancer, neurodegenerative diseases, metabolic syndrome, hypertension, and inflammation [2]. However, the effects of short-term fasting on physiological and psychological responses are not well understood.

Little and equivocal information is available regarding the effects of short-duration fasting on mood and cognition. There is evidence of adverse effects [4, 5] or no effects [6, 7] of total or near-total calorie deprivation on mood and cognition. Moreover, we are not aware of any previous randomized studies of the effect of a 48 h, zero-calorie diet on cognition and mood.

Recent years have shown growing interest in the association between autonomic cardiovascular function and the brain [8, 9] and in the observation that resting heart rate variability (HRV) as a marker of cardiovascular autonomic function [10] can reflect the activity of flexible and integrative neural networks allowing maximal organism accommodation in the face of rapidly changing environmental demands [9]. It has been suggested that there is a relationship between the vagally mediated high-frequency- (HF-) HRV and cognitive functioning (specifically with prefrontal-cortex-related tasks) [8] and between HF-HRV and mood [11, 12]. A recent study reported that 48 h fasting decreased HRV; however, the HF component of HRV was not affected [3]; thus, it can be expected that 48 h fasting would not affect mood and cognition.

It is noteworthy that 48 h fasting increased sympathetic activity and was indicated as stress [3, 13]. The observed common hypotensive response during the first days of fasting is not due to a decreased sympathetic activity [13]. It is well established that the brain is a target of stress and that the prefrontal cortex is the brain area that is most sensitive to its effects [14, 15]; the prefrontal complex is responsible for highest-order cognitive abilities, such as executive functions [14, 16]. Interestingly, sympathetic nerve activity does not influence cerebral blood flow [17]; thus, it has no effect on brain activity. Lieberman et al. [7] reported that 48 h of near-total calorie deprivation decreased glucose level. It is well known that brain activity and function depend on glucose and that continuous delivery of its main source of energy from the blood is required [18]. Thus, it can be expected that increased stress (i.e., an increased activity of the sympathetic nervous system) and decreased glucose levels would decrease brain activity and would adversely affect mood and cognitive performance, which are specifically related to prefrontal-cortex-mediated functions.

Recent evidence suggests that chronic resistance training decreases stress levels (see Huang et al. [19] for review). However, the stress-induced autonomic cardiovascular response [19] and its effect on cognition and mood remain debatable. Weight lifting is becoming more common because of an increased awareness of the need to maintain individual physical fitness [20]. Thus, the main aim of this study was to estimate the effect of a 48 h, zero-calorie diet with water provided* ad libitum* on autonomic function, brain activity, cognition, and mood in amateur weight lifters. Furthermore, based on the sensitivity to stress in different brain regions [14], we considered that it was important to compare performance in prefrontal-cortex-mediated executive (working memory, mental flexibility, and shifting set) and hippocampus-mediated nonexecutive (visuospatial discrimination and spatial orientation ability) tasks.

## 2. Material and Methods

### 2.1. Subjects

Thirteen men were assessed for eligibility. The criteria for inclusion were (1) age 20–30 years; (2) amateur weight lifters (regular resistance training for at least 1 year); (3) nonsmokers; (4) no involvement in any weight loss diet; (5) no medications that could affect response to fasting; and (6) no evidence of any acute or chronic bodily or mental disease, eating disorders, and past trauma of head. In total, 9 volunteers with more than 3 years of weightlifting training experience met the inclusion criteria and agreed to participate in this study. Their physical characteristics are presented in Table 1. Written informed consent was obtained from all participants after explanation of all details of the experimental procedures and the associated discomforts and risks. All procedures were approved by the Human Research Ethics Committee and were conducted according to the guidelines of the Declaration of Helsinki. Subjects were in self-reported good health, as confirmed by medical history and physical examination.

### 2.2. Procedures

One week before the experiment, the subjects were familiarized with the laboratory setting and with the experimental procedures used for cognitive testing. Before the start of the experiment, the subjects were instructed to sleep for ≥8 h the night before the experiment and to refrain from ingesting alcoholic beverages, caffeine, and sedating antihistamines for ≥48 h and from heavy exercise for at least 24 h before the experiment.

The experiment consisted of two sessions that were performed in the morning at 8h00. During the first session, the participant arrived at the laboratory after overnight fasting (8–12 h), to complete baseline measurements. On arrival at the laboratory, an anthropometric measurement was performed, electrocardiogram (ECG) electrodes were attached to the chest, and a functional near-infrared spectroscopy (fNIRS) probe was attached to the frontal areas of the forehead. The participant was asked to rest in a sitting position for 10 min in a quiet, semidimmed room. Using fNIRS, resting brain activity was recorded during the last 5 min, and, using ECG, the resting cardiovascular autonomic response was recorded during the last 2 min. Subsequently, the light in the room was switched on, control measurements of blood pressure (BP) and glucose level were performed, and the participants completed the Brunel Mood Scale and identified their hunger and fullness level. The participants were then seated at a table in a well-lit room, and cognitive testing was performed. Mental effort investment and motivation during the testing were rated. The participants rested at least 1 day before starting the second session, which consisted of a 48 h, zero-calorie diet with water provided* ad libitum*, followed by the performance of experimental measurements in the same order as that used in the first sessions (i.e., before fasting).

### 2.3. Measurements

#### 2.3.1. Anthropometric Measurements

The participant's weight and body mass index (BMI) (TBF-300 body composition scale; Tanita, UK Ltd., West Drayton, UK) were estimated while the subjects were seminude (shorts and T-shirts).

#### 2.3.2. Analysis of Blood Samples

The blood glucose levels were determined in capillary blood samples (0.3 *μ*L) using a glucose analyzer (Glucocard X-mini plus meter; Arkray, Japan).

#### 2.3.3. Assessment of Autonomic Cardiovascular Function

A computerized ECG analytical system (Kaunas-load; Institute of Cardiology, LUHS, Kaunas, Lithuania) was employed for synchronous standard 12-lead ECG recording and analysis of autonomic function parameters. In the time domain that reflects general heart rate variability (HRV), the standard deviation of normal-to-normal intervals (SDNN; estimate of overall HRV) and the root mean square of successive differences (RMSSD; estimate of short-term components of HRV) were assessed. In the frequency domain that measures the more specific contribution of the autonomic nervous system branch, we used the fast Fourier transform to assess low-frequency (LF; estimates sympathetic and parasympathetic activity) and high-frequency (HF; estimates parasympathetic activity) powers in absolute and normalized units [10]. In addition, the frequency and time domain variables were logarithmically transformed (Ln) to correct the skewness of distribution. In addition, heart rate (HR) (i.e., ECG R-R interval) was assessed, and indirect arterial BP measurements were taken from the upper arm using a sphygmomanometer (Sanaphon; Riester, Jungingen, Germany) and a standard-size arm cuff.

#### 2.3.4. Assessment of Brain Activity

The fundamentals of fNIRS have been described in detail elsewhere [21]. An assessment of brain activity was performed on a continuous wave system (fNIR Imager 1100, fNIR Devices LLC, Potomac, Maryland, USA) using a flexible 16-optode probe set (consisting of 10 photodetectors and four light emitters each using light with a wavelength of 730 and 850 ± 15 nm). The sensor has a temporal resolution of 500 ms/scan with 2.5 cm source-detector separation, allowing for a penetration depth of approximately 1.25 cm and 16 measurement locations on a rectangular grid covering the forehead region, which was designed to monitor dorsal and inferior frontal cortical areas. The COBI Studio software was used for data acquisition. The signals of all channels were verified before recording. Data analysis was performed using the fNIRSoft analysis software (BIOPAC Systems Inc., USA). Oxygenated hemoglobin (OxyHb) and deoxygenated hemoglobin (DeoxyHb) values from raw data were calculated by solving the modified Beer-Lambert equation. Data were filtered to remove physiological and other artifacts. The changes in OxyHb, DeoxyHb, and their sum, that is, total hemoglobin (TotalHb), were acquired from all participants in all 16 channels and the data were averaged.

#### 2.3.5. Assessment of Cognitive Function

Cognitive function was assessed using the computerized automated neuropsychological assessment metric Version 4 (ANAM-4; Vista Life Sciences, USA), which is a reliable screening instrument that was designed for repeated evaluations [22]. The test battery was performed in ~12 min and included the following chosen tests (in random order) to measure task accuracy (percent correct responses) and mean response time (mean reaction time).

The* Matching Grids Task (MGT)* measures visuospatial discrimination [23]. During this test, two 4 × 4 grids are displayed side by side on the screen; however, one 4 × 4 pattern is rotated. The participant is instructed to indicate as quickly as possible if the grids are exactly the same, with the exception of a possible rotation, and press the left mouse button and the right mouse button if the grids are different. This test consisted of 20 trials.

The* Manikin Task (MT)* measures spatial orientation ability [22]. During this test, the figure of a man is presented holding a ball in one hand and a cube on the other hand, and a ball or a cube is displayed at the bottom of the screen. The man's figure appears in various orientations: standing upright or upside down and either facing toward or away from the test taker. The participant is instructed to indicate as quickly as possible which of the man's hands is holding the object displayed at the bottom of the screen and to press the left mouse button if the hand is the left one and the right mouse button if the hand is the right one. This test consisted of 32 trials.

The* Memory Search Task (MST) *is an adaptation of Sternberg's [24] memory search/serial reaction time task, which measures verbal working memory [22, 23]. During this test, a string of six letters is presented for memorization. The participant is instructed to press the space bar once he has memorized the letter string; then, it disappears from view and individual letters are presented one at a time. The participant is instructed to indicate as quickly as possible whether the letter belongs to the memorized set and press the left mouse button for memory set letters and the right mouse button for letters not included in the memory set. This test consisted of 40 trials.

The* Mathematical Processing Task (MPT) *measures working memory [22]. During this test, an arithmetic problem requiring an addition and subtraction of three single-digit numbers is displayed (e.g., “5 − 2 + 3 = ”). The participant is instructed to indicate as quickly as possible and to press the left mouse button if the answer to the equation is greater than 5 or press the right mouse button if the answer is less than 5. The correct answer may be any number from 1 to 9, except 5. This test consisted of 20 trials.

The* Two-Choice Reaction Time Task (TCRTT)* measures the ability to shift the mental set (mental flexibility) [22]. During this test, one of two stimuli is presented on the screen (“*∗*” or “o”) with a variable interstimulus interval. The participant is instructed to respond as quickly as possible by pressing the left mouse button each time the “*∗*” stimulus is presented or the right mouse button each time the “o” stimulus is presented. This test consisted of 40 trials.

The* Switching Task (ST) *measures the ability of mental flexibility and shifting set [22]. This task is a combination of MT and MPT. The MT is located on the left side of the computer screen and the MPT is located on the right side of the computer screen, and the user is directed by means of a red arrow at the bottom of the screen to respond to the problem on the left or on the right. Responses are entered using the keyboard, with the left hand used for the MT and the right hand used for MPT. This test consisted of 64 trials.

#### 2.3.6. Self-Assessment Questionnaires

The invested mental effort during cognitive tasks was measured using the 9-point subjective rating scale developed by Paas [25], which ranges from 1 (very, very low mental effort) to 9 (very, very high mental effort).

Based on scale used in a previous study [26], motivation with regard to the cognitive task performance was assessed on a visual analog scale (VAS) ranging from 1 (not motivated at all) to 10 (extremely motivated) on a 10 cm long horizontal line. Current hunger and fullness were also assessed on a VAS ranging from 0 (“I am not hungry at all/not at all full”) to 100 (“I have never been more hungry/totally full”) [27].

Current mood (“how do you feel right now?”) before and after fasting was assessed using the Brunel Mood Scale [28]. This questionnaire contains 24 items divided into six respective subscales: anger, confusion, depression, fatigue, tension, and vigor. The items are answered on a 5-point scale ranging from 0 (not at all) to 4 (extremely), and each subscale, with four relevant items, can achieve a raw score in the range of 0 to 16.

### 2.4. Statistical Analysis

To assess the effect of fasting on measured variables, a paired sample *t*-test was performed. Pearson correlation coefficients (*r*) were used to identify relationships between variables. If a significant fasting effect or correlation was found, the bootstrap method was used to confirm the significance of results, and corrected level of significance was presented. The simulated differences were based on 1000 bootstrap samples. The magnitude of fasting effects within the group was estimated by Cohen's effect size (ES) test. Cohen defined 0.2–0.3 as a small effect, ~0.5 as a medium effect, and >0.8 as a large effect. The level of significance was set at *p* < 0.05. Data are presented as means and standard deviations (SD). Statistical analysis was carried out using SPSS v.21.0 (IBM Corp., Armonk, NY, USA).

## 3. Results

### 3.1. Characteristics of the Subjects

The characteristics of the subjects are described in Table 1. Fasting significantly decreased weight (*p* = 0.003, ES = 0.24), BMI (*p* = 0.001, ES = 0.20), and glucose level (*p* = 0.012, ES = 0.14).

### 3.2. Effects of Fasting on Cardiovascular Autonomic Function

Table 2 presents resting BP and short-term HRV values before and after fasting. Fasting significantly decreased resting HR (*p* = 0.044, ES = 0.25) and systolic BP (*p* = 0.039, ES = 0.64), whereas HRV parameters were not affected by it.

### 3.3. Effects of Fasting on Resting Brain Activity

Table 3 presents fasting-induced hemodynamic response on resting frontal cortex. Fasting significantly decreased (*p* = 0.040, ES = 0.64) OxyHb, whereas DeoxyHb and TotalHb were not affected by it. A significant positive correlation was found between glucose level and OxyHb (*r* = 0.55, *p* = 0.47).

### 3.4. Effects of Fasting on Cognitive Performance

The motivation to perform cognitive tasks was 8.39 (1.83) before fasting and 6.72 (2.82) after fasting. The mental effort during cognitive tasks was indicated as being “neither low nor high mental effort” (5.00 (1.22) and 4.78 (1.09) before and after fasting, resp.). Motivation and mental effort did not differ compared with those recorded before fasting.  Table 4 presents cognitive performance values before and after fasting. Fasting significantly decreased reaction time in the TCRTT (*p* = 0.049, ES = 0.56) and ST tasks (*p* = 0.004, ES = 0.98), whereas performance on other tasks was not affected. Significant positive correlations were found between OxyHb and reaction time in the MPT (*r* = 0.60, *p* = 0.024) and OxyHb and reaction time in the ST (*r* = 0.53, *p* = 0.048), and tendency of negative correlation was found between OxyHb and accuracy of the TCRTT (*r* = −0.50, *p* = 0.070), whereas other cognitive tasks were not significantly correlated with OxyHb.

### 3.5. Effects of Fasting on Mood State and Subjective Feelings of Hunger and Fullness

**Figure 1:**
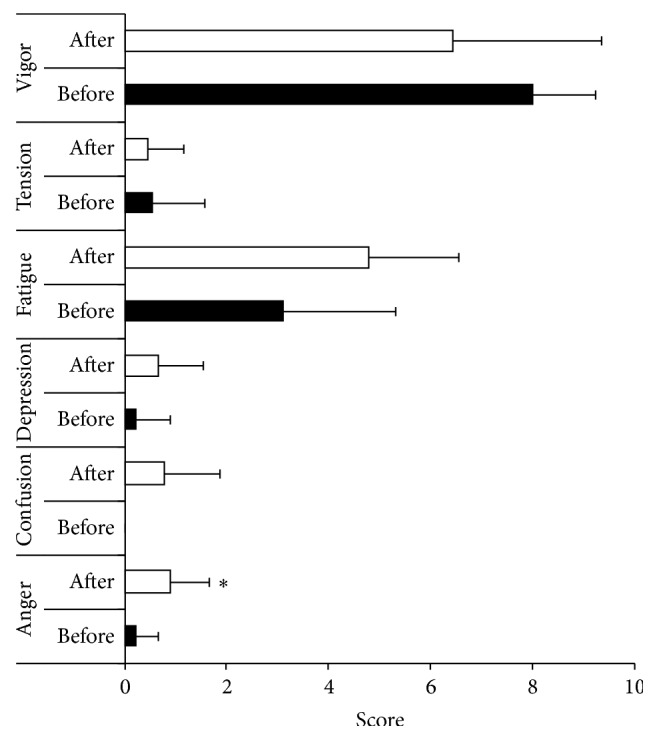
Mood state before and after fasting. Data are presented as mean (standard deviation). ^*∗*^ *p* < 0.05, compared with before fasting.

Figure 1 presents mood state values before and after fasting. Fasting significantly increased (*p* = 0.039, ES = 1.09) anger, whereas other mood states were not affected significantly by it. We observed a slight tendency toward an increase in confusion (*p* = 0.089) and fatigue (*p* = 0.090). Fasting significantly increased (*p* = 0.045, ES = 1.71) the feeling of hunger, from 42.8 (23.6) to 77.2 (16.8), and decreased (*p* = 0.001, ES = 3.10) the feeling of fullness, from 40.6 (12.1) to 1.7 (3.5). A significant positive correlation was found between glucose level and fullness (*r* = 0.58, *p* = 0.015) and a negative correlation was detected between glucose level and hunger (*r* = −0.49, *p* = 0.046). The feeling of fullness correlated negatively with anger (*r* = −0.49, *p* = 0.049) and the feeling of hunger correlated positively with fatigue (*r* = 0.62, *p* = 0.008) and negatively with vigor (*r* = −0.53, *p* = 0.028). There was no significant correlation between the subjective feelings of hunger and fullness and other mood states.

## 4. Discussion

The aim of the present study was to determine the effect of 48 h fasting on autonomic function, brain activity, cognition, and mood in amateur weight lifters. We observed that HR and BP were reduced after fasting, whereas all 2 min HRV indices were unchanged after fasting. Furthermore, we found decreased mood and improved prefrontal-cortex-mediated cognitive functions such as mental flexibility and set shifting after fasting. In contrast, hippocampus-related cognitive performance was not affected by fasting. In accordance with decreased glucose level, resting brain activity was decreased. To our knowledge, this is the first study to examine physiological and psychological changes after 48 h fasting in resistance-trained men.

In accordance with Andersson et al. [13], we observed a hypotensive response (decreased BP) to 48 h fasting. In addition, we observed a decrease in HR, which is in accordance with a predominant increase in the activity of the parasympathetic nervous system [29, 30]. In contrast Chan et al. [31] observed an increase in HR after 72 h of fasting. The contradictory results of HR might be related to different fasting duration used in previous study. The limitation of the present study is that we did not assess neuroendocrine response to 48 h fasting. Andersson et al. [13] did not find significant changes in norepinephrine level after 48 h fasting; thus, it can be expected that catecholamine level was not affected in the current study. It is noteworthy that ketones are produced in response to a low glucose level [32] and that the major ketone body, *β*-hydroxybutyrate, inhibits the sympathetic nervous system [33]. Sympathetic activity could be suppressed to save energy as a survival mechanism, resulting in a decrease in HR [33, 34].

In contrast to the results of Mazurak et al. [3], HRV was not affected in the current study, which might suggest that 48 h total fasting is not associated with an increased risk for cardiac events [35] in amateur weight lifters. Interestingly, we observed that short-term fasting resulted in higher parasympathetic activity; however HF-HRV estimating parasympathetic activity [10] was not changed. The results of unchanged HRV might be related to small sample size (*n* = 9) and testing conditions. There is evidence that changes associated with fasting are more pronounced with a stress challenge to the cardiac regulatory system during tilt testing than during resting activity [3]. Moreover, we are not aware of test-retest reliability of resting 2 min HRV data recorded in sitting position. Thus, the results of HRV should be interpreted with caution.

In contrast to our expectations and previous studies [4, 6, 7], we observed a fasting-induced increase in prefrontal-cortex-related cognitive flexibility tasks. Working memory, visuospatial discrimination, and spatial orientation abilities were not affected. It is well established that brain function depends on glucose level [18]. We observed that four out of nine subjects exhibited a reduced glucose level, to clinically hypoglycemic concentrations (<3.5 mmol/L) [36]. However, an increase in ketone level may substitute the low glucose level after fasting [32, 37]. Moreover, the metabolism of ketone bodies is more efficient than that of glucose, leading to increased available energy for adenosine triphosphate synthesis [37].

In accordance with our expectation and glucose decrease [18], we observed that the total 48 h, zero-calorie diet decreased resting brain activity (decreased OxyHb), which was associated with a decreased glucose level. The resting brain state has been challenged by functional neuroimaging studies that showed that a set of brain regions displays elevated activity at rest and revealed a systematically decreased activity during cognitively demanding tasks [38]. Some authors speculate on the existence of an active organized baseline mode of brain function [39]. We observed that a lower resting OxyHb was strongly associated with enhanced prefrontal-cortex-related tasks performance, but not with hippocampus-related task performance. We are not aware if there was any change in the hippocampus baseline mode; thus, further studies are needed.

We did not find any fasting-induced HF-HRV changes, which could explain the improved cognitive performance observed and its relationship with executive and nonexecutive fasting-induced changes (see Jennings et al. [8] for review). In a previous study reported by Crippa et al. [40], it was observed that the injection of acetylcholine into the prefrontal cortex of rats mediated a hypotensive response. Thus, it might be suggested that the decrease in BP observed in the current study was associated with an increase in acetylcholine level in the brain. Recent studies have provided support for a role of cortical acetylcholine in attentional orienting and stimulus discrimination, and the prefrontal cortex is one of the most important areas in these processes [41]. It is noteworthy that increased cognitive flexibility may also emerge because of slightly increased anger, which was shown to be specifically associated with improved selective attention [42].

In accordance with our expectation and a previous study by Uher et al. [5], we found that the total 48 h, zero-calorie diet adversely affected mood. We did not find any fasting-induced HF-HRV changes; however in present study parasympathetic activity increase (HR and BP decrease) was observed. Interestingly, it contrasts previous studies [11, 12, 43] showing that negative emotions (e.g., anger) are associated with the parasympathetic activity decrease and sympathetic activity increase. It was suggested that mood changes affects autonomic system response. However, in current study evoked parasympathetic activity increase was associated with fasting-induced physiological rather than the emotional state.

It is well established that fasting decreases blood glucose level [7], which has long been regarded as a biochemical marker of hunger [44]. We found a significant negative correlation between glucose and hunger and a positive correlation between glucose and fullness. Moreover, an increased subjective feeling of hunger and a decreased subjective feeling of fullness were associated with decreased vigor and increased anger and fatigue. We observed that fasting significantly increased anger, which is a basic emotion that is important to overcome in the anticipation of desired goals (e.g., food-seeking behavior during starvation) [2, 45]. The effect of mood state observed in the current study is consistent with the effect of anger on enhanced selective attention reported in a previous study [42]. In contrast, the recent study by Lieberman et al. [7] reported that a 48 h near-total calorie diet has no effect on mood state. The authors suggested that the double-blind, placebo-controlled procedures prevented subjective expectations and mood degradation in the calorie-restricted state [7]. According to the associations between glucose, subjective feelings, and mood states observed in our study, we suggest that the differences recorded between studies might be explained by differences in glucose decrease and availability (60 versus 0 g) and by different satiety levels caused by varying calorie availability (313 versus 0 kcal). Moreover, it is established that glucose may alter mood [46] and, in some individuals, we observed a reduction in glucose level, to clinically hypoglycemic concentrations, which is in contrast to previous research [7], which found that glucose level was within the clinically acceptable range (>3.5 mmol/L).

Of note, our subjects were healthy, motivated amateur athletes with a history of several years in sports. Thus, the findings associated with the 48 h total calorie deprivation cannot be applied to other populations, such as patients or nonphysically active subjects. Furthermore, there is evidence of differences in stress responses [47, 48] and stress resilience [15] between men and women. As a consequence, our results can only be generalized to men, and whether the effects found here also pertain to women has yet to be established.

## 5. Conclusions

Our study showed that acute 48 h fasting resulted in higher parasympathetic activity and decreased resting frontal brain activity in amateur weight lifters. Furthermore, we found increased anger and improved prefrontal-cortex-mediated cognitive functions, such as mental flexibility and set shifting, after fasting. In contrast, hippocampus-related cognitive performance was not affected by total calorie deprivation. It is well established that not only resistance-trained individuals but also aerobically trained individuals exhibit an attenuated response to stressors [19]. Thus, additional research is needed to determine the effectiveness of and role played by different exercise and physical activity forms (aerobic training versus resistance training) in short-term fasting.

## Figures and Tables

**Table 1:** Characteristics of the subjects before and after fasting.

Parameter	Before	After
	Fasting	
Age, yr	25.9 (4.1)	
Height, cm	181.0 (4.5)	
Mass, kg	89.3 (12.8)	86.8 (12.0)^*∗*^
BMI, kg/m^2^	27.3 (3.8)	26.4 (3.7)^*∗*^
Glucose, mmol/L	4.81 (0.65)	3.74 (0.60)^*∗*^

Data are presented as mean (standard deviation). ^*∗*^ *p* < 0.05, compared with before fasting.

BMI, body mass index.

**Table 2:** Resting blood pressure and 2 min heart rate variability parameters before and after fasting.

Parameter	Before fasting	After fasting
Blood pressure		
SBP (mmHg)	120.2 (7.1)	115.6 (7.3)^*∗*^
DPP (mmHg)	76.1 (5.5)	73.9 (6.0)
Heart rate variability		
HR (bpm)	80.8 (17.8)	76.2 (19.5)^*∗*^
SDNN (ms)	45.3 (27.3)	52.8 (28.4)
SDNN (ln (ms))	3.68 (0.54)	3.83 (0.57)
RMSSD (ms)	21.5 (20.6)	22.78 (16.5)
RMSSD (ln (ms))	2.74 (0.79)	2.89 (0.73)
LF (ms^2^)	720.2 (579.9)	785.6 (454.3)
HF (ms^2^)	695.0 (629.8)	770.0 (618.3)
LF (ln (ms^2^))	6.34 (0.69)	6.52 (0.57)
HF (ln (ms^2^))	6.27 (0.73)	6.33 (0.89)
LF (nu)	51.8 (9.1)	54.3 (12.4)
HF (nu)	48.2 (9.1)	45.7 (12.4)

Data are presented as mean (standard deviation). ^*∗*^ *p* < 0.05, compared with before fasting.

SBP, systolic blood pressure; DBP, diastolic blood pressure; HR, heart rate; SDNN, standard deviation of normal to normal interval; RMSSD, root mean square of the successive differences; LF, low frequency; HF, high frequency.

**Table 3:** Resting frontal cortex hemodynamic response before and after fasting.

Parameter	Before fasting	After fasting
OxyHb (*μ*M)	1.09 (0.44)	0.33 (0.83)^*∗*^
DeoxyHb (*μ*M)	0.02 (0.52)	−0.11 (0.96)
TotalHb (*μ*M)	1.10 (0.73)	0.01 (1.40)

Data are presented as mean (standard deviation). ^*∗*^ *p* < 0.05, compared with before fasting.

OxyHb, oxygenated hemoglobin; DeoxyHb, deoxygenated hemoglobin; TotalHb, total hemoglobin.

**Table 4:** Cognitive performance before and after fasting.

Task	Reaction time (ms)		Accuracy (%)	
	Before	After	Before	After
	Fasting		Fasting	
MGT	1385.6	1450	94.4	95.6
	(319.6)	(305.3)	(6.8)	(4.6)
MT	1565.1	1529.7	96.2	87.8
	(413.6)	(448.2)	(5.6)	(27.3)
MST	903.7	863.2	93.3	96.1
	(163.4)	(165.7)	(8.0)	(4.9)
MPT	2057.2	1902.4	94.4	96.7
	(717.9)	(378.6)	(5.3)	(5.0)
TCRTT	493.6	453.0	95.8	97.2
	(87.0)	(57.0)^*∗*^	(3.8)	(2.3)
ST	2278.6	1937.5	91.4	96.3
	(370.5)	(325.1)^*∗*^	(7.8)	(2.6)

Data are presented as mean (standard deviation). ^*∗*^ *p* < 0.05, compared with before fasting.

MGT, Matching Grids Task; MT, Manikin Task; MST, Memory Search Task; MPT, Mathematical Processing Task; TCRTT, Two-Choice Reaction Time Task; ST, Switching Task.
